# Professional Notes

**Published:** 1887-12-10

**Authors:** Geo. W. Potter


### PROFESSIONAL NOTES.

By GEO. W. POTTER, M.D.

"VOLVULUS and intussusception," says Dr. Randolph Winslow, of Maryland, "offer the most favourable conditions for recovery, and yet they are attended with a very large mortality. But in the case of internal constrictions from adhesions or bands, whether from congenital defects or pathological changes, a cure by the process of nature is of such rarity that it ought not to come into consideration, and the patient, unless relieved by art, is almost inevitably doomed to death. Unfortunately"—and this is the crux of the difficulty—"it is not possible to reach a certain diagnosis in all cases; but this very difficulty is one of the strongest arguments for opening the abdomen, both as a diagnostic and a curative measure. I certainly think severe pain, obstinate constipation, and stercoraceous vomit are a congeries of symptoms of sufficient gravity not only to justify but to demand imperatively the performance of laparotomy. Six years ago a case of intestinal obstruction, probably from volvulus, or possibly from an impacted gall-stone, came under the care of the writer, which was relieved by medical means. The benefit lasted, however, but a short time, when symptoms of obstruction again set in, and only terminated with the life of the patient. I proposed an operation, but was over-ruled by the consulting surgeon. This case made a deep impression upon my mind, and I determined not to let a similar case die on my hands without at least offering the patient the chance of relief by laparotomy or colotomy. The importance of an early recognition of intestinal obstruction cannot be over-estimated, and with its recognition comes the duty of being prepared to give the patient every possible chance of recovery. These cases come when least expected, and demand prompt attention, and usually not much time is allowed the practitioner for consultation or reference. He must often act upon his own responsibility, promptly and boldly, or the golden opportunity will be lost."

---

MR. JONATHAN HUTCHINSON, on the other hand, as decidedly opposes early surgical interference in cases of intestinal obstruction, and thinks it also of little value in those which have been some time in existence. He advocates what he calls *abdominal taxis*, under an anæsthetic. By abdominal taxis Mr. Hutchinson means "a thorough kneading of the abdomen, with inversion of the patient, shaking him, tossing him in a blanket, and a variety of rough gymnastics, the object being to dislodge the bowel or untwist the volvulus. At the same time he uses large enemata and laxative medicines. If these means do not succeed, he waits and keeps the patient on a low diet, and gives opium or belladonna internally, and subsequently repeats the abdominal taxis. He reports a series of interesting cases in which a cure was effected by these means. He favours laparotomy as a last and most desperate resort." (*International Journal of the Medical Sciences.*) One such case the present writer saw with Mr. Hutchinson. Abdominal taxis, with very large enemata were the means resorted to, and afterwards low diet, with opium and belladonna in full doses; the blanket-tossing was, however, omitted. The patient, a young man of twenty-four, recovered well. In the course of four or five weeks later he developed typhoid fever. From that also he recovered; but during convalescence symptoms of lung abscess showed themselves, and the patient, after passing safely through two of the most formidable dangers known to medicine, seemed as if he must die after all. He was seen by Dr. Bristowe and others. Finally, after having been more than seventeen weeks in bed, he rallied, and ultimately got quite well. Altogether he was absent from business about twelve months. He is now a strong man of thirty. The present writer's experience in other similar cases tends to confirm Mr. Hutchinson's practice.

DIAPHRAGMATIC PLEURISY is an affection seldom recognised by the practitioner, though it is perhaps more frequent than is generally believed. Some points brought out by Dr. Frank Donaldson, jun., in a study of the subject (*International Journal*), are well worthy of reproduction and consideration. The disease seems to have been known to the ancients in a general way. They describe it under the names *phrenitis*, *paraphrenitis*, *diaphragmitis*, etc. Hippocrates used the term "phrenitis," but as designating a febrile state only, and not as indicating any disease of the diaphragm itself. Oelguinetus and Tralian seem to have known true phrenitis, for they speak of the distinct type of the respiratory movement. Galen also knew diaphragmitis as a distinct affection, and in a chapter entitled "Phrenitis from Inflammation of the Brain, and that from Inflammation of the Diaphragm," describes the delirium which follows inflammation of the brain, of the lungs, and of the pleura, and remarks that inflammation of no other organ causes such continued delirium as that of the diaphragm. Boerhaave gives a complete description under the name "paraphrenitis," recognising it as an inflammation of the serous covering. Roy, in describing the "risus sardonicus," declares it to be a constant symptom of inflammation of the diaphragmatic pleura. Laennec describes the disease under the general head of "circumscribed pleurisies." Andral was the first to publish definite observations under the name of "diaphragmatic pleurisy," and to show that the affection might be primary or secondary. The disease seems to be most frequent in the adult and in women. It is rare in children. The *secondary* form is most often caused by tuberculosis, and is then invariably fatal. A summary of the symptoms is as follows:—The onset is variable: usually there is a chill of greater or less severity, followed by fever and perspiration; intense pain in the side, and constriction of the lower part of the chest soon follow the chill; the pains are characteristic, and are to be referred to the terminal filaments of the phrenic nerves. They are what Hermil calls "douleurs par propogation." The pains extend over a large surface, over the whole hypochondriac region and over both flanks, to the inferior dorsal region behind, following the line of the costal insertions of the diaphragm, and often along the border of the sternum, and under the lower insertion of the sterno-cleido-mastoid muscle, and over the shoulders and neck. The pains may be spontaneous, but are always evoked by pressure or by increased respiratory movements, hiccough, and vomiting. According to De Mussy, the following are the particular seats where pain may be evoked on pressure: Over the epigastrium, at the points of insertion of the tendons of the diaphragm; in the last intercostal space behind, near the spine; along the course of the phrenic nerve; and finally, at what he calls *le bouton diaphragmatique*, where the pain is always especially great. This spot is at a point one or two fingers' breadth from the middle line, on a level with the tenth rib. Pressure in the intercostal spaces always gives rise to pain. As to the frequency of pain in any given spot, that in the hypochondriac region is oftenest present. A particular point always to be borne in mind in the diagnosis is the *attitude* of the patient. This is said to be quite characteristic: the *body is always bent forward*, the dorsal position being impossible, and the *hands are applied to the sides* in order to *fix the diaphragm* as much as possible. The expression is one of great anxiety and suffering; the *risus sardonicus* is often present, though it is not, as was formerly supposed, a positively diagnostic symptom. *The most distinctive and prominent symptom is the frequency and shallowness of the respirations.* Respiration is entirely costal, and limited to the upper part of the chest. It may be as frequent as 80 or 100. The immobility of the diaphragm is a direct cause of the retraction of the hypochondriac region, which is invariably present. Galen and the ancients considered this retraction to be pathognomonic. The immobility, however, is rarely complete, and is mostly unilateral. Auscultation gives negative results. The cough is frequent, dry, and painful; the expectoration slight and thin. Hiccough is a distressing symptom. There is sometimes violent pain when a bolus of food passes the diaphragm. The delirium is often very marked. The course of the disease is variable. In uncomplicated cases the maximum intensity is soon reached, and the symptoms subside as rapidly as they appeared. Secondary diaphragmatic pleurisies are usually fatal. In simple cases the treatment must be the same as for ordinary pleurisies; that is to say, movements must be reduced to a minimum, and such other measures taken as would be employed in inflammations of serous surfaces.

